# Weight status and psychosomatic complaints in Swedish adolescent boys and girls: does family support play a buffering role?

**DOI:** 10.1186/s12889-024-20517-6

**Published:** 2024-10-31

**Authors:** Jasmin Venäläinen, Sara Brolin Låftman, Jonas Landberg

**Affiliations:** Department of Public Health Sciences, Stockholm University, Stockholm, SE-106 91 Sweden

**Keywords:** Weight status, Overweight, Obesity, Psychosomatic complaints, Family support, Adolescents, HBSC

## Abstract

**Background:**

Psychosomatic complaints have increased among adolescents in recent decades, as have overweight and obesity rates. Both of these trends are regarded as public health concerns. However, the associations between weight status and psychosomatic complaints are not yet clear, necessitating further research. The aim of the present study was to investigate the associations between weight status and psychosomatic complaints in Swedish adolescent boys and girls, as well as to explore the potential buffering effect of family support.

**Methods:**

The data was obtained from the cross-sectional Swedish Health Behaviour in School-aged Children (HBSC) study conducted in 2017/18, which involved 3,135 students aged 11, 13, and 15 years. Weight status was based on self-reported information on weight and height, which allowed for the calculation of body mass index (BMI) and the categorisation of participants into three groups: non-overweight, overweight, and obese. Psychosomatic complaints were assessed based on information regarding the frequency of eight different complaints, which were summed into an index. Family support was measured using three items describing the level of perceived emotional support, and an index was created, which was dichotomised into low and high family support. Gender stratified linear regression models were run to examine the associations between weight status and psychosomatic complaints. Age and family affluence were included as covariates. Interaction terms were included to evaluate whether family support moderated the main association.

**Results:**

Obesity was associated with higher levels of psychosomatic complaints in both boys and girls when compared to being non-overweight (boys: b = 2.56, 95% CI 0.32, 4.79; girls: b = 3.35, 95% CI 0.77, 5.94), while being overweight did not show any statistically significant associations with the outcome (boys: b = 0.21, 95% CI -0.72, 1.15; girls: b = 0.78, 95% CI -0.42, 1.98). In girls, a statistically significant interaction effect between family support and weight status was observed (*p* = 0.031), indicating that family support buffered against psychosomatic complaints in girls with obesity. No statistically significant interaction was found for boys (*p* = 0.642).

**Conclusions:**

The findings of this study highlight the importance of public health initiatives aimed at preventing childhood obesity. They also underscore the significant role of family support in reducing psychosomatic complaints among adolescents with obesity. Further research is necessary to gain a deeper understanding of these relationships.

**Supplementary Information:**

The online version contains supplementary material available at 10.1186/s12889-024-20517-6.

## Introduction

Overweight and obesity represent a growing global public health concern, contributing to the development of various physical and psychological health issues [1]. In children and adolescents, overweight and obesity increase the risk of experiencing poor health outcomes in adulthood [2]. While numerous studies have shed light on the physical consequences of overweight and obesity, there has been relatively less research focusing on their psychological implications in adolescents [3]. Existing research has linked overweight and obesity to higher levels of stress, anxiety and depression in adolescents [4, 5]. Furthermore, a recent study using German data [6] found that higher BMI scores in children and adolescents were associated with increased behavioural difficulties and lower health-related quality of life. However, there have been fewer studies examining potential protective factors. The current study focuses on the relationship between weight status and psychosomatic complaints in adolescent boys and girls, as well as the potentially moderating (i.e., buffering) influence of family support.

Individuals’ identity develops throughout the life course; however, adolescence is a critical phase when identity formation is particularly pronounced [7]. The concept of weight stigma refers to negative attitudes, beliefs, and discrimination based on an individual’s weight, which can be associated with the way people see themselves and how others see them, impacting one’s body image [8]. People with overweight or obesity may experience weight stigma, as it is frequently directed at their physical appearance, particularly their weight and body size [9]. Individuals who are overweight or obese may also be labeled as lazy and unhealthy, which can lead to discrimination against them. Weight stigma can be encountered in various situations and from different sources, including the workplace, family, health care professionals, and school. Experiencing weight stigma can result in adverse psychological and physical health outcomes, particularly among adolescents [9]. Children and young people with overweight or obesity might face bullying, social exclusion, and discrimination due to weight stigma [10]. These experiences can trigger negative emotions, such as anxiety, among adolescents suffering from overweight or obesity. More significantly, weight stigma can induce stress, lower self-esteem, and contribute to a negative body image. Over an extended period, these factors may lead to mental health problems [9].

Psychosomatic complaints encompass physical and psychological symptoms, such as headaches and nervousness, that do not originate from any specific disease. These complaints commonly manifest during the transition from adolescence to adulthood [11]. Poor mental well-being may contribute to experiencing psychosomatic complaints, and according to the World Health Organization (WHO) [12], mental health problems are the leading health issue among adolescents. Perceived stress is a significant factor that previous studies have found to be associated with psychosomatic complaints among adolescents [13, 14]. While psychosomatic complaints can occur individually, they often tend to accumulate [15, 16]. These complaints can disrupt individuals’ daily lives, affecting their ability to function normally (due to pain, not being able to sleep, etc.), ultimately reducing their overall quality of life [17]. Experiencing psychosomatic complaints during youth can increase the likelihood of these complaints persisting into adulthood, increasing the risks of other health-related problems and more severe psychological issues [15, 18]. Moreover, psychosomatic symptoms in adolescence have been demonstrated to predict the development of depressive and anxiety disorders in adulthood [19, 20].

Overweight and obesity may be connected to psychosomatic complaints through various mechanisms, including psychological factors like stress and anxiety related to weight stigma [21], as well as physical consequences such as musculoskeletal problems [22]. A cohort study that examined the weight status of Swedish adolescents and their experience of psychosomatic complaints found that being overweight or obese was associated with higher levels of such complaints [21]. In a study that focused on adolescents in Europe and North America, conducted by Whitehead et al. [23], it was revealed that when adolescents perceived themselves as overweight, their levels of psychosomatic complaints increased, particularly among girls.

High family support and positive family relationships have been found to promote adolescents’ well-being by increasing life satisfaction and enhancing their quality of life [24]. They contribute to strengthened self-esteem and self-worth [25]. Furthermore, high family support can assist adolescents in managing stress and protecting against adverse health outcomes, such as anxiety [26]. This aligns with the stress-buffering model of social support [27], suggesting that a supportive family environment can shield adolescents from or help them cope with stressors. As such, a plausible hypothesis would be that family support may act as a buffering factor in reducing psychosomatic complaints among adolescents with overweight or obesity.

While psychosomatic complaints are prevalent in both genders, prior studies have consistently indicated that girls tend to report more complaints compared to boys [11, 13, 18, 21, 28]. Moreover, women who are overweight or obese tend to report a higher occurrence of mental health problems (e.g., depressive symptoms), compared to their male counterparts [29, 30]. Consequently, when investigating the connections between adolescents’ weight status and psychosomatic complaints, conducting gender-specific analyses is relevant.

Age can play a significant role in adolescents’ weight and their levels of psychosomatic complaints. During adolescence, individuals undergo physical and psychological changes. Physical changes include growth in height and weight, which can impact their body image [31]. Additionally, as adolescents mature, they become more independent and start making their own decisions, which may influence their health, potentially leading to behaviours like increased consumption of fast food [32]. Studies have also indicated that physical activity levels tend to decrease as adolescents grow older [32, 33]. Furthermore, individuals from more affluent backgrounds generally exhibit better behaviours than those with low family affluence, contributing to their overall well-being [33, 34]. Low family affluence has also been linked to higher rates of overweight and obesity [2, 33]. Therefore, in an analysis of weight status and psychosomatic complaints, it is essential to consider both age and family affluence.

### Aim of the study

The overall objective of this study was to explore the associations between adolescent weight status, psychosomatic complaints, and family support among Swedish boys and girls. Specifically, we aimed to determine whether adolescents who are overweight or obese report higher levels of psychosomatic complaints compared with those with normal weight, and whether family support can act as a buffer against such an association. The research questions were:

1. Are overweight and obesity associated with higher levels of psychosomatic complaints in adolescent boys and girls, even when adjusting for age and family affluence?
2. Does high family support reduce the strength of these possible associations?

### Methods

#### Data material

The data was obtained from the Swedish Health Behaviour in School-aged Children (HBSC) survey of 2017/18. HBSC is a quantitative cross-national survey conducted in collaboration with the World Health Organization (WHO) and has been collected every four years since 1983/84. The survey includes information collected amongst 11-, 13- and 15-year-old boys and girls. The main objective of the HBSC survey is to investigate adolescents’ overall well-being, their health behaviours, living environments and social contacts. The surveys cover a wide range of topics, including adolescent sexual health, alcohol and drug usage, family and friend relationships, socio-economic environment, health habits and body image. Adolescents complete the questionnaires independently [35, 36].

In the Swedish HBSC survey of 2017/18, a cluster sampling method was used with classes being randomly selected. In all, 450 schools were selected for the survey, and 213 schools participated, with one class in each school, i.e., a school-level response rate of 47%. In total, 4,294 Swedish adolescents participated in the survey of whom 1,181 11-year-olds, 1,452 13-year-olds and 1,661 15-year-olds, corresponding to a student-level response rate of 89% [37]. The Swedish 2017/18 data file from the HBSC Open Access Data Portal (https://hbsc.org/data/) included 4,185 individuals. After excluding cases with missing values on the variables that were used for this study, the analytical sample consisted of 3,135 individuals (1,528 boys and 1,607 girls), corresponding to 74.9% of the total sample. Most of internal non-response was related to the questions on weight and height, which were used to assess weight status (see Supplementary Material, Table S1).

### Measurements

Weight status was measured by adolescents’ self-reports about their height and weight, which were computed into BMI (body mass index), using the international IOTF (International Obesity Task Force) cut-off points [38]. Implausible height and weight values were classified as missing data by the HBSC Data Management Centre [39]. The original variable included 4 categories: thinness, normal weight, overweight, and obese. For the current study, thinness and normal weight were collapsed into the same category, after checking that the thinness and normal weight categories had similar associations with the outcome, and the main purpose of this study being to investigate adolescents with overweight or obesity. The collapsed category was named non-overweight, and the exposure variable thus included 3 categories: non-overweight, overweight, and obese. While direct measurements of height and weight are preferable, self-reported values are considered reliable proxies across adolescent subpopulations, regardless of age, sex, or race/ethnicity [40].

Psychosomatic complaints were measured by the question: “In the last 6 months: how often have you had the following?” The eight items were headache, stomach ache, backache, feeling low, feeling irritability or bad temper, feeling nervous, having difficulties with sleeping and feeling dizzy. The response categories were: (1) “about every day,” (2) “more than once a week,” (3) “about every week,” (4) “about every month,” and (5) “rarely or never”. The variables were reversely coded, so that higher points would indicate higher complaints. An index ranging between 8 and 40 points was created, in which higher values corresponded to higher levels of complaints. Internal consistency was high (Cronbach’s α = 0.83). The measure has been used previously [23, 28, 41] and has demonstrated good test-retest reliability, unidimensionality, and external validity [23]. For an additional check, we split the index into psychological and somatic complaints, respectively, each with the range 4–20, and used these as two separate outcome variables. Psychological complaints included feeling low, feeling irritability or bad temper, feeling nervous, and having difficulties with sleeping, whereas somatic complaints included headache, stomach ache, backache, and feeling dizzy.

Family support was operationalised by three items from the Multidimensional Scale of Perceived Social Support [42] focusing on emotional support: “I get the emotional help and support I need from my family”, “I can talk about problems with my family”), and “My family really tries to help me”. The response categories were on a 7-point Likert scale from 1 = Very strongly disagree to 7 = Very strongly agree. An index was created with the range 3–21, where higher values corresponded to higher family support. Internal consistency was high (Cronbach’s α = 0.90). Next, the index was dichotomised into the categories: low support and high support. The cut off was made so that approximately the highest 75 pct would indicate high support (resulting in values 3–17 indicating low support and values 18–21 indicating high support).

Gender included two categories: boys and girls.

Age included three categories: 11-, 13- and 15-year-olds, corresponding to the Swedish school grades 5, 7 and 9.

Family affluence was assessed by the Family Affluence Scale, based on information on the family’s number of bathrooms, computers, and cars, if the adolescent has their own bedroom, if the household has a dishwasher, and how many times the family has been on holidays/travelabroad during the past 12 months [43]. We used a relative measure of family affluence which included three categories: lowest 20 pct, medium 60 pct, and highest 20 pct.

### Statistical analysis

First, descriptive statistics were explored for the total study sample and stratified by gender. Next, we examined distributions of the outcome (psychosomatic complaints) and the covariates (age and family affluence) by categories of the exposure (weight status), stratified by gender. Chi-square tests and ANOVAs were used to evaluate differences by weight status. Then, a series of linear regression models were run to investigate the associations between weight status and psychosomatic complaints, stratified by gender. Unstandardised b coefficients with 95% confidence intervals are presented. Crude analyses included one independent variable at a time. Model 1 included the exposure (weight status) adjusted for age and family affluence. Model 2 added family support. Model 3 included an interaction term between weight status and family support, which was evaluated by a Wald test. The results of the interaction analyses were visualised by means of margins plots (adjusted predictions by Stata’s “margins” command). In all models, robust standard errors were estimated to account for the hierarchical nature of the data, with students being nested in classes. All analyses were performed with Stata version 17.0 [44].

### Large language models (LLMs)

ChatGPT was used to check the grammar, proofread the text and provide clarifications.

## Results

Table 1 presents descriptive statistics of the study sample, consisting of 1,528 boys (48.7%) and 1,607 girls (51.3%). In the study sample, 86.6% of the adolescents were non-overweight, 11.4% were overweight, and 2.0% were classified as obese. Specifically, among boys, the proportions were 84.5%, 13.1%, and 2.4%, respectively; while among girls, the proportions were 88.5%, 9.7%, and 1.7%, respectively. The distribution of age groups was somewhat uneven with 24.5% 11-year-olds, 33.3% 13-year-olds, and 42.2% 15-year-olds. The lowest family affluence group contained 13.6% of the sample, the medium group 68.4% and the highest group 18.0% of the sample. Following the dichotomisation of family support, 75.2% of adolescents belonged to the high support group (78.7% among boys and 71.9% among girls), whereas 24.8% were in the low support group (21.3% among boys and 28.1% among girls). In the total sample, the mean score for psychosomatic complaints was 18.73 on a scale ranging from 8 to 40, with girls (20.42) having a higher mean score than boys (16.95). Distributions of all separate symptoms are presented in the Supplementary Material, Table S2.

**Table 1:** Descriptives of the study sample

	All (*n* = 3,135)		Boys (*n* = 1,528)		Girls (*n* = 1,607)	
	n	%	n	%	n	%
Weight status						
Non-overweight	2,715	86.6	1,292	84.5	1,423	88.5
Overweight	356	11.4	200	13.1	156	9.7
Obese	64	2.0	36	2.4	28	1.7
Age						
11 years	767	24.5	384	25.1	383	23.8
13 years	1,044	33.3	511	33.4	533	33.2
15 years	1,324	42.2	633	41.4	691	43.0
Relative family affluence (FAS)						
Lowest 20ptc	427	13.6	204	13.4	233	13.9
Medium 60ptc	2,145	68.4	1,047	68.5	1,098	68.3
Highest 20ptc	563	18.0	277	18.1	286	17.8
Family support						
Low	777	24.8	325	21.3	452	28.1
High	2,358	75.2	1,203	78.7	1,155	71.9
	Mean	s.d.	Mean	s.d.	Mean	s.d.
Psychosomatic complaints	18.73	6.65	16.95	5.88	20.42	6.88

As shown in Table 2, weight status did not show any statistically significant differences between age groups. In both boys and girls, weight status varied significantly by relative family affluence (*p* < 0.01 in both genders). The proportion of adolescents who were non-overweight was highest among those with highest family affluence, whereas the proportion of adolescents with overweight were highest among those with lowest family affluence. For boys, obesity was most common among those with medium family affluence, and for girls among those with lowest family affluence. There was no statistically significant association between weight status and family support for boys (*p* > 0.05), whereas for girls, low levels of family support were more common among those who were overweight or obese (*p* < 0.05). Psychosomatic complaints were significantly associated with weight status in a graded manner in both genders, with the lowest levels among adolescents who were non-overweight and the highest levels among those who were obese (*p* < 0.05 in both genders).

**Table 2:** Distributions of outcome and covariates by exposure. Differences in the distributions by exposure groups assessed with χ^2^ tests and ANOVAs

	Boys (*n* = 1,528)				Girls (*n* = 1,607)			
	Non-overweight	Overweight	Obese		Non-overweight	Overweight	Obese	
	%	%	%	χ^2^	%	%	%	χ^2^
Age								
11 years	85.4	12.8	1.8		87.7	11.5	0.8	
13 years	82.8	14.3	2.9		89.1	8.3	2.6	
15 years	85.5	12.3	2.2	2.41	88.6	9.8	1.6	6.97
Relative family affluence (FAS)								
Lowest 20ptc	77.9	20.1	2.0		81.2	16.6	2.2	
Medium 60ptc	84.6	12.8	2.6		89.3	8.7	1.9	
Highest 20ptc	89.2	9.0	1.8	13.69**	91.3	8.0	0.7	16.73**
Family support								
Low	84.0	13.2	2.8		85.2	12.2	2.6	
High	84.7	13.1	2.2	0.32	89.9	8.7	1.4	7.74*
	Mean	Mean	Mean	ANOVA(F)	Mean	Mean	Mean	ANOVA(F)
Psychosomatic complaints	16.86	17.08	19.42	3.37*	20.29	21.07	23.64	4.03*

**p < 0.01 *p < 0.05

Results from linear regression analyses of weight status and psychosomatic complaints among boys are presented in Table 3. The crude analyses show that boys who were overweight did not report higher levels of psychosomatic complaints than boys who were non-overweight (b = 0.21, 95% CI -0.72, 1.15), whereas those who were obese did (b = 2.56, 95% CI 0.32, 4.79). Furthermore, there was a statistically significant difference in psychosomatic complaints between boys who were overweight and those who were obese (*p* = 0.049) (not presented in Table). There was a difference by age in that 15-year-old boys reported more psychosomatic complaints compared with 11-year-olds (b = 1.16, 95% CI 0.24, 2.09). No statistically significant differences in boys’ psychosomatic complaints by relative family affluence were detected. Boys who reported high family support reported significantly lower levels of psychosomatic complaints compared with those who reported low family support (b=-3.52, 95% CI -4.32, -2.72). The associations between obesity and psychosomatic complaints remained statistically significant when controlling for age and family affluence (Model 1) and when adding family support (Model 2). The estimate of family support was statistically significant also in Model 2. The difference in psychosomatic complaints between 11- and 15-year-old boys remained in Model 1 but turned non-significant in Model 2 after accounting for family support. The interaction between weight status and family support was tested in Model 3, but turned out to be non-significant (*p* = 0.642).

**Table 3:** Results from linear regression analyses of psychosomatic complaints regressed on weight status and covariates, among boys. Regression coefficients and 95% confidence intervals (95% CI) with robust standard errors. *n* = 1,528

	Crude		Model 1		Model 2		Model 3	
	b	95% CI	b	95% CI	b	95% CI	b	95% CI
Weight status								
Non-overweight (ref.)	0.00	-	0.00	-	0.00	-	0.00	-
Overweight	0.21	-0.72, 1.15	0.26	-0.68, 1.19	0.28	-0.62, 1.18	0.43	-1.67, 2.52
Obese	2.56*	0.32, 4.79	2.56*	0.32, 4.81	2.44*	0.33, 4.55	4.49	-0.91, 9.90
Age								
11 years (ref.)	0.00	-	0.00	-	0.00	-	0.00	-
13 years	0.04	-0.89, 0.98	-0.02	-0.97, 0.92	-0.16	-1.06, 0.75	-0.16	-1.07, 0.75
15 years	1.16*	0.24, 2.09	1.11*	0.17, 2.04	0.82	-0.08, 1.72	0.81	-0.09, 1.71
Family affluence								
Lowest 20ptc (ref.)	0.00	-	0.00	-	0.00	-	0.00	-
Medium 60ptc	0.50	-0.45, 1.45	0.45	-0.50, 1.41	0.72	-0.24, 1.68	0.76	-0.20, 1.72
Highest 20ptc	-0.04	-1.26, 1.18	0.05	-1.16, 1.26	0.55	-0.66, 1.75	0.58	-0.63, 1.78
Family support								
Low (ref.)	0.00				0.00	-	0.00	-
High	-3.52***	-4.32, -2.72			-3.46***	-4.25, -2.66	-3.36***	-4.19, -2.53
Interaction between weight status and family support								
Overweight*High family support							-0.19	-2.63, 2.25
Obese*High family support							-2.73	-8.46, 3.00
Wald test							*p* = 0.642	

***p < 0.001 *p < 0.05

Crude analyses include one variable at a time

Model 1 includes weight status, age, and family affluence

Model 2 includes weight status, age, family affluence, and family support

Model 3 includes weight status, age, family affluence, family support, and the interaction between weight status and family support

Table 4 presents results from linear regression analyses of weight status and psychosomatic complaints among girls. The crude analyses show that, compared with girls who were non-overweight, those who were overweight did not report higher levels of psychosomatic complaints (b = 0.78, 95% CI -0.42, 1.98). However, those who were obese had higher levels of psychosomatic complaints (b = 3.35, 95% CI 0.77, 5.94). The difference in psychosomatic complaints between girls who were overweight and obese was borderline significant (*p* = 0.057) (not presented in Table). There was a graded association in psychosomatic complaints by age, with 11-year-old girls displaying the lowest levels; higher levels in 13-year-olds (b = 2.17, 95% CI 1.21, 3.12); and even higher in 15-year-olds (b = 4.17, 95% CI 3.28, 5.05). Girls with high family affluence had lower levels of psychosomatic complaints compared with those with low family affluence (b=-1.52, 95% CI -2.75, -0.29). High family support was inversely associated with psychosomatic complaints (b=-5.72, 95% CI -6.50, -4.93). The association between obesity and psychosomatic complaints attenuated slightly but remained statistically significant when adjusting for age and family affluence (Model 1), and weakened even further when adding family support (Model 2). Family support remained associated with psychosomatic complaints (b=-5.28, 95% CI -6.05, -4.50). The difference in psychosomatic complaints by family affluence, observed in the crude model, became non-significant after adjusting for other variables in Models 1 and 2. However, the graded association by age remained statistically significant across all models. The interaction between weight status and family support was added in Model 3. The Wald test revealed a statistically significant interaction term (*p* = 0.031), suggesting that high family support attenuated the association between obesity and psychosomatic complaints in girls.

**Table 4:** Results from linear regression analyses of psychosomatic complaints regressed on weight status and covariates, among girls. Regression coefficients and 95% confidence intervals (95% CI) with robust standard errors. *n* = 1,607

	Crude		Model 1		Model 2		Model 3	
	b	95% CI	b	95% CI	b	95% CI	b	95% CI
Weight status								
Non-overweight (ref.)	0.00	-	0.00	-	0.00	-	0.00	-
Overweight	0.78	-0.42, 1.98	0.75	-0.45, 1.96	0.36	-0.73, 1.44	0.28	-1.69, 2.24
Obese	3.35*	0.77, 5.94	3.08*	0.52, 5.65	2.35*	0.20, 4.49	5.90**	2.47, 9.32
Age								
11 years (ref.)	0.00	-	0.00	-	0.00	-	0.00	-
13 years	2.17***	1.21, 3.12	2.15***	1.18, 3.12	1.75***	0.89, 2.61	1.74***	0.89, 2.60
15 years	4.17***	3.28, 5.05	4.16***	3.27, 5.05	3.42***	2.57, 4.27	3.41***	2.56, 4.26
Family affluence								
Lowest 20ptc (ref.)	0.00	-	0.00	-	0.00	-	0.00	-
Medium 60ptc	-0.83	-1.87, 0.21	-0.98	-2.01, 0.05	-0.66	-1.58, 0.27	-0.66	-1.59, 0.27
Highest 20ptc	-1.52*	-2.75, -0.29	-1.13	-2.33, 0.07	-0.41	-1.51, 0.69	-0.46	-1.57, 0.64
Family support								
Low (ref.)	0.00	-			0.00	-	0.00	-
High	-5.72***	-6.50, -4.93			-5.28***	-6.05, -4.50	-5.16***	-5.96, -4.36
Interaction between weight status and family support								
Overweight*High family support							0.13	-2.45, 2.71
Obese*High family support							-6.18**	-10.78, -1.58
Wald test							*p* = 0.031	

***p < 0.001 **p < 0.01 *p < 0.05

Crude analyses include one variable at a time

Model 1 includes weight status, age, and family affluence

Model 2 includes weight status, age, family affluence, and family support

Model 3 includes weight status, age, family affluence, family support, and the interaction between weight status and family support

**Fig. 1:**
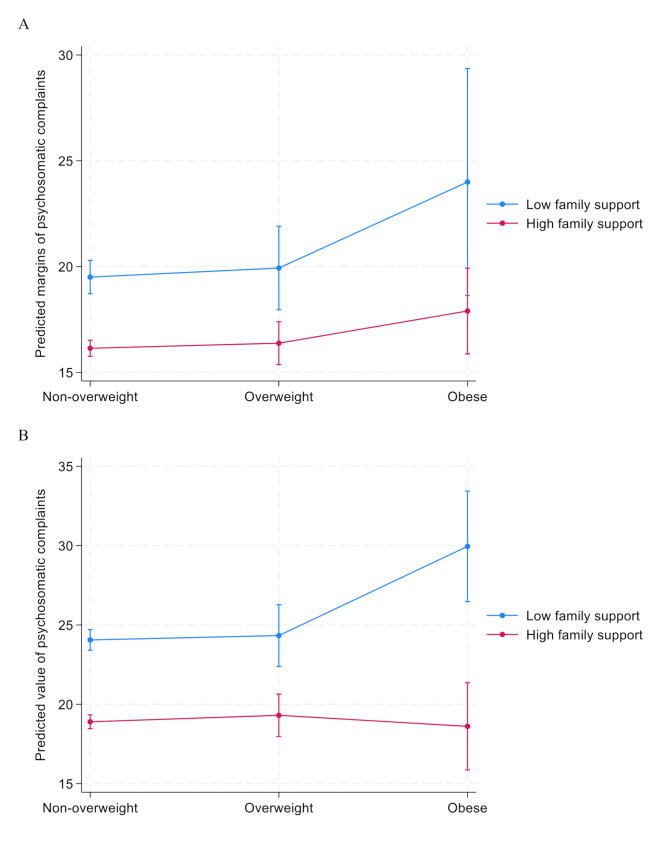
**A**. Predicted margins of psychosomatic complaints by weight status and family support (adjusting for age and relative family affluence), among boys. *n* = 1,528. **B**. Predicted margins of psychosomatic complaints by weight status and family support (adjusting for age and relative family affluence), among girls. *n* = 1,607

The interplay between weight status and family support in relation to psychosomatic complaints is visualised in Fig. 1A (for boys) and Fig. 1B (for girls). In particular for girls, the association between obesity and psychosomatic complaints was affected by the level of family support. A similar but less pronounced (and not statistically significant) tendency was seen among boys.

To further illustrate these findings, results from linear regression analyses of the associations between weight status and psychosomatic complaints, stratified by gender and level of family support, are presented in the Supplementary Material, Table S3. For boys, there were no statistically significant associations between overweight or obesity and psychosomatic complaints, regardless of whether they had high or low family support. In contrast, for girls, obesity was associated with higher levels of psychosomatic complaints among those with low family support (b = 5.90, 95% CI 2.64, 9.16), but not among those with high family support (b=-0.19, 95% CI -2.95, 2.57).

The descriptive analyses presented in Table 2 revealed a statistically significant difference in weight status by family support among girls, but not among boys. Specifically, the prevalence of overweight and obesity was higher among girls reporting low family support compared to those reporting high family support. To scrutinise this association further, we conducted additional gender-stratified multivariable logistic regression analyses (not presented in Table) with overweight or obesity (vs. non-overweight) as the dependent variable and family support as the independent variable. Controlling for age and relative family affluence, the analyses confirmed that girls with high family support had a lower likelihood of being overweight or obese (OR 0.67, 95% CI 0.47–0.95), whereas no such association was found in boys (OR 1.00, 95% CI 0.71–1.41).

Finally, we performed gender-stratified linear regression analyses using psychological and somatic complaints as two separate outcome variables, with results presented in the Supplementary Material, Tables S4-S7. The results show largely similar patterns across both outcomes.

## Discussion

The aim of this study was to explore the connections between adolescent weight status, psychosomatic complaints, and family support among boys and girls in Sweden. Our specific focus was to investigate whether adolescents classified as overweight or obese reported higher levels of psychosomatic complaints compared to those who were non-overweight. Additionally, we aimed to determine if family support had the potential to act as a buffer against such an association.

The first research question aimed to determine whether there was an association between overweight and obesity and increased levels of psychosomatic complaints in adolescent boys and girls, even after adjusting for age and family affluence. The results indicated that adolescents who were obese reported more complaints compared to those who were non-overweight, with no such association found among adolescents who were overweight. This pattern held true for both boys and girls. The finding that obesity was associated with higher levels of psychosomatic complaints is largely consistent with previous research. Studies of adolescents have shown that overweight and obesity are associated with higher levels of stress, anxiety and depression [4, 5]. Furthermore, a cohort study conducted with Swedish individuals aged 12–19 years [21] found a connection between overweight/obesity and higher levels of psychosomatic complaints. However, while that study combined overweight and obesity into a single category, our results did not show a statistically significant association between overweight and psychosomatic complaints, but only between obesity and psychosomatic complaints. One possible interpretation is that overweight, being relatively common, may not be as strongly associated with body shaming. Moreover, a study on mental health in children and adolescents with overweight or obesity based on German data [6] included somatoform complaints as one of the outcomes, measured in a similar way to psychosomatic complaints in our study. The associations between BMI and somatoform complaints pointed in the expected direction but did not reach statistical significance.

One potential mechanism in the link between obesity and psychosomatic complaints is weight stigma. This can lead to negative physical and psychological health outcomes, including a negative self-image, low self-esteem and a negative body image, potentially resulting in a negative social identity [3, 9]. Experiences such as social exclusion and bullying, driven by negative stereotyping, may contribute to psychological issues that, in turn, manifest as psychosomatic complaints [8, 10]. Relatedly, it is possible that adolescents compare their bodies to those of their peers but also to unrealistic body shapes portrayed in social media and other sources. Such comparisons may be especially stressful for those who are obese [23]. Finally, while genetic and physiological factors may contribute to both obesity and depression [4], it is possible that these factors are also linked to psychosomatic complaints. Taken together, however, it is important to note that there are relatively few studies that have explored the associations between weight status and psychosomatic health among adolescents. Therefore, further research is necessary to enhance our understanding of the connections between weight and psychosomatic complaints. Studies investigating both mediating and moderating effects are crucial for elucidating the mechanisms involved and identifying potential protective and exacerbating factors. Additionally, research utilising both survey and qualitative data could offer valuable insights.

The second research question addressed whether family support could act as a buffer against psychosomatic complaints in adolescents with overweight or obesity. In both boys and girls, high family support exhibited a significant negative association with psychosomatic complaints, consistent with previous research [24, 45, 46]. Additionally, a statistically significant buffering effect was observed in girls with obesity, while no statistically significant buffering effect was found in boys. This finding for girls may be interpreted in the context of the stress-buffering model of social support, which suggests that emotional and social support can aid individuals in coping with stressors and lead to better health outcomes by reducing their stress levels [27]. One possible interpretation of the gender-specific findings in this study is that girls may place a higher value on and be more affected by family support compared to boys. The estimates of family support were indeed stronger for girls than for boys in this study. This interpretation aligns with prior research, which has reported a stronger negative association between parental support and psychosomatic complaints among girls than among boys [46]. Additionally, maternal support has been found to be more strongly linked to reduced levels of depressive symptoms among girls compared to boys [47].

It is also noteworthy that additional analyses in this study identified a difference in weight status based on family support among girls, but not boys. In particular, girls with high family support had a lower likelihood of being overweight or obese, whereas no such association was found in boys. This suggests a potential direct effect of family support on weight status among girls, where higher support is associated with a lower likelihood of overweight and obesity. This effect in girls could potentially be driven by increased parental involvement and the promotion of healthy behaviours, including fostering healthy eating habits and physical activity.

### Strengths and limitations

The main strength of the current study lies in its use of BMI to assess weight status, alongside a validated measure of psychosomatic complaints that has been used in prior studies [48]. Another merit is the large national sample, providing a comprehensive view of the well-being of Swedish boys and girls. However, there are also some limitations to consider. One of these pertains to missing values in the data. While the full sample includes information from 4,185 participants, the final sample size decreased to 3,135 after excluding cases with missing values for the variables used in this study. A significant number of participants did not answer questions related to their height, weight, or experiences of psychosomatic complaints. This was particularly prominent among 11-year-olds, possibly because, as the youngest participants in the study, they might not have been certain about their exact height and weight. Additionally, although self-reported measures of weight and height are regarded as reasonable proxies [40], the validity of the BMI measure may be compromised if participants struggled to report their weight and height accurately. Another limitation of this study is possibility of non-response bias [49]. Some of the adolescents who were invited to participate were absent from school on the day they were scheduled to complete the survey. This absence from school could be due, in part, to psychosomatic complaints. However, it is more likely that such potential non-response bias may have led to an underestimation of the effects rather than the reverse.

Furthermore, because the data in this study is cross-sectional, it does not allow for the establishment of causality [49]. Obesity could potentially serve as a causal factor for psychosomatic complaints, but these complaints could also lead to weight gain. For instance, adolescents experiencing psychosomatic complaints may find it difficult to engage in physical activity, which could increase their risk of becoming overweight or obese. Alternatively, physical activity could act as a confounding factor that influences both weight status and psychosomatic complaints. Regular physical activity can help maintain a healthy weight and protects against overweight and obesity, while also improving overall well-being, potentially reducing levels of psychosomatic complaints. Similarly, healthy eating habits could act as a confounder. A healthy diet may protect against excessive weight gain and enhance overall well-being, potentially decreasing the levels of psychosomatic complaints [50]. Future studies should consider the inclusion of physical activity and other potential confounders.

### Implications of the findings

The findings of this study make valuable contributions to our understanding of the relationship between obesity and psychosomatic complaints in adolescents. First, considering the strong association between obesity and elevated psychosomatic complaints, it is relevant to prioritise public health strategies aimed at preventing childhood and adolescent obesity. Second, the findings point at the relevance of incorporating psychosocial interventions in treatment of overweight and obesity [6]. Third, since high family support has the potential to reduce psychosomatic complaints in adolescents with obesity, it is important to encourage and support parents or caregivers in developing and enhancing their supportive skills, and to ensure they are present and available for their children. This can significantly enhance the well-being of adolescents suffering from obesity and reduce the likelihood of poor health in adulthood. Additionally, initiatives to strengthen family support may also have an indirect effect on psychosomatic complaints by helping reduce the prevalence of overweight and obesity among girls.

## Conclusions

This study showed that adolescents with obesity reported higher levels of psychosomatic complaints, compared with their non-overweight peers. The association was found for boys and girls alike. The significant link between obesity and increased levels of psychosomatic complaints underscores the importance of obesity prevention and the implementation of public health initiatives to address this issue. Additionally, the finding that family support had a positive impact in reducing psychosomatic complaints among girls with obesity – while no such buffering effect was found among boys – suggests that girls may benefit more from family support than boys. Moreover, strong family support appears to have a direct impact on lowering the prevalence of overweight and obesity among girls.

## Electronic Supplementary Material

Below is the link to the electronic supplementary material.

Supplementary Material 1

## Data Availability

Data are available from the HBSC Data Management Center, University of Bergen. Please see: https://www.uib.no/en/hbscdata/113290/open-access.

## Abbreviations

95% CI: 95% Confidence Interval
ANOVA: Analysis of Variance
BMI: Body Mass Index
HBSC: Health Behaviour in School-aged Children
OR: Odds Ratio
WHO: World Health Organization
