# Supplementary material for: Weight status and psychosomatic complaints in Swedish adolescent boys and girls: does family support play a buffering role?
Source: BMC Public Health. 2024 Oct 31;24:3024. doi: 10.1186/s12889-024-20517-6 (PMC11529216; doi:10.1186/s12889-024-20517-6)
Supplement: Supplementary file 1 — Supplementary Material 1 [file 12889_2024_20517_MOESM1_ESM.docx]

**Supplementary Material. Table S1.** Descriptives of the full sample.

|  | All  (n=4,185) | | Boys  (n=2,081) | | Girls  (n=2,104) | |
| --- | --- | --- | --- | --- | --- | --- |
|  | n | % | n | % | n | % |
| Weight status |  |  |  |  |  |  |
| Non-overweight | 2,911 | 86.1 | 1,417 | 84.1 | 1,494 | 87.9 |
| Overweight | 400 | 11.8 | 229 | 13.6 | 171 | 10.1 |
| Obese | 72 | 2.1 | 38 | 2.3 | 34 | 2.0 |
| *Missing* | *802* | *-* | *397* | *-* | *405* | *-* |
| Age |  |  |  |  |  |  |
| 11 years | 1,174 | 28.1 | 603 | 29.0 | 571 | 27.1 |
| 13 years | 1,423 | 34.0 | 715 | 34.3 | 708 | 33.7 |
| 15 years | 1,588 | 38.0 | 763 | 36.7 | 825 | 39.2 |
| *Missing* | *0* | *-* | *0* | *-* | *0* | *-* |
| Relative family affluence (FAS) |  |  |  |  |  |  |
| Lowest 20ptc | 628 | 15.7 | 305 | 15.4 | 323 | 15.9 |
| Medium 60ptc | 2,688 | 67.0 | 1,323 | 66.7 | 1,365 | 67.2 |
| Highest 60ptc | 696 | 17.3 | 354 | 17.9 | 342 | 16.9 |
| *Missing* | *173* | *-* | *99* |  | *74* | *-* |
| Family support |  |  |  |  |  |  |
| Low support | 999 | 24.7 | 432 | 21.6 | 567 | 27.6 |
| High support | 3,050 | 75.3 | 1,565 | 78.4 | 1,485 | 72.4 |
| *Missing* | *136* | *-* | *84* | *-* | *52* | *-* |
|  | Mean | s.d | Mean | s.d. | Mean | s.d. |
| Psychosomatic complaints | 18.63 | 6.68 | 16.89 | 5.98 | 20.31 | 6.89 |
| *Missing* | *176* |  | *115* |  | *61* |  |

**Supplementary Material. Table S2.** Distributions of all separate psychosomatic complaints, by gender.

|  | Boys  (n=1,528) | | Girls  (n=1,607) | |
| --- | --- | --- | --- | --- |
|  | n | % | n | % |
| Headache |  |  |  |  |
| About every day | 44 | 2.9 | 152 | 9.5 |
| More than once a week | 151 | 9.9 | 257 | 16.0 |
| About every week | 255 | 16.7 | 325 | 20.2 |
| About every month | 470 | 30.8 | 445 | 27.7 |
| Rarely or never | 608 | 39.8 | 428 | 26.6 |
| Backache |  |  |  |  |
| About every day | 70 | 4.6 | 93 | 5.8 |
| More than once a week | 103 | 6.7 | 139 | 8.7 |
| About every week | 183 | 12.0 | 176 | 11.0 |
| About every month | 343 | 22.4 | 384 | 23.9 |
| Rarely or never | 829 | 54.3 | 815 | 50.7 |
| Stomach ache |  |  |  |  |
| About every day | 32 | 2.1 | 107 | 6.7 |
| More than once a week | 104 | 6.8 | 248 | 15.4 |
| About every week | 210 | 13.7 | 282 | 17.6 |
| About every month | 481 | 31.5 | 603 | 37.5 |
| Rarely or never | 701 | 45.9 | 367 | 22.8 |
| Feeling dizzy |  |  |  |  |
| About every day | 34 | 2.2 | 79 | 4.9 |
| More than once a week | 79 | 5.2 | 155 | 9.7 |
| About every week | 162 | 10.6 | 193 | 12.0 |
| About every month | 356 | 23.3 | 350 | 21.8 |
| Rarely or never | 897 | 58.7 | 830 | 51.7 |
| Feeling low |  |  |  |  |
| About every day | 59 | 3.9 | 204 | 12.7 |
| More than once a week | 156 | 10.2 | 308 | 19.2 |
| About every week | 199 | 13.0 | 348 | 21.7 |
| About every month | 433 | 28.3 | 403 | 25.1 |
| Rarely or never | 681 | 44.6 | 344 | 21.4 |
| Feeling irritable |  |  |  |  |
| About every day | 107 | 7.0 | 254 | 15.8 |
| More than once a week | 332 | 21.7 | 515 | 32.1 |
| About every week | 406 | 26.6 | 406 | 25.3 |
| About every month | 455 | 29.8 | 294 | 18.3 |
| Rarely or never | 228 | 14.9 | 138 | 8.6 |
| Feeling nervous |  |  |  |  |
| About every day | 40 | 2.6 | 136 | 8.5 |
| More than once a week | 190 | 12.4 | 333 | 20.7 |
| About every week | 353 | 23.1 | 425 | 26.5 |
| About every month | 538 | 35.2 | 433 | 26.9 |
| Rarely or never | 407 | 26.6 | 280 | 17.4 |
| Having sleeping difficulties |  |  |  |  |
| About every day | 143 | 9.4 | 252 | 15.7 |
| More than once a week | 253 | 16.6 | 279 | 17.4 |
| About every week | 260 | 17.0 | 314 | 19.5 |
| About every month | 323 | 21.1 | 306 | 19.0 |
| Rarely or never | 549 | 35.9 | 456 | 28.4 |

**Supplementary Material. Table S3.** Results from linear regression analyses of psychosomatic complaints regressed on weight status, age, and family affluence, stratified by gender and level of family support. Regression coefficients and 95% confidence intervals (95% CI) with robust standard errors.

|  | Boys with low  family support  (n=325) | | Boys with high  family support  (n=1,203) | | Girls with low  family support  (n=452) | | Girls with high  family support  (n=1,155) | |
| --- | --- | --- | --- | --- | --- | --- | --- | --- |
|  | b | 95% CI | b | 95% CI | b | 95% CI | b | 95% CI |
| Weight status |  |  |  |  |  |  |  |  |
| Non-overweight (ref.) | 0.00 | - | 0.00 | - | 0.00 | - | 0.00 | - |
| Overweight | 0.41 | -1.66, 2.49 | 0.24 | -0.81, 1.29 | 0.08 | -1.88, 2.04 | 0.40 | -1.04, 1.83 |
| Obese | 4.41 | -0.85, 9.67 | 1.78 | -0.28, 3.84 | 5.90*** | 2.64, 9.16 | -0.19 | -2.95, 2.57 |

***p<0.001 **p<0.01 *p<0.05

**Supplementary Material. Table S4.** Results from linear regression analyses of psychological complaints regressed on weight status and covariates, among boys. Regression coefficients and 95% confidence intervals (95% CI) with robust standard errors. n=1,528

|  | Crude | | Model 1 | | Model 2 | | Model 3 | |
| --- | --- | --- | --- | --- | --- | --- | --- | --- |
|  | b | 95% CI | b | 95% CI | b | 95% CI | b | 95% CI |
| Weight status |  |  |  |  |  |  |  |  |
| Non-overweight (ref.) | 0.00 | - | 0.00 | - | 0.00 | - | 0.00 | - |
| Overweight | 0.16 | -0.41, 0.74 | 0.21 | -0.37, 0.78 | 0.22 | -0.32, 0.77 | 0.15 | -1.07, 1.36 |
| Obese | 0.92 | -0.30, 2.14 | 0.93 | -0.30, 2.16 | 0.85 | -0.34, 2.04 | 0.61 | -2.11, 3.34 |
| Age |  |  |  |  |  |  |  |  |
| 11 years (ref.) | 0.00 | - | 0.00 | - | 0.00 | - | 0.00 | - |
| 13 years | -0.03 | -0.55, 0.48 | -0.08 | -0.60, 0.45 | -0.17 | -0.66, 0.33 | -0.17 | -0.66, 0.33 |
| 15 years | 0.75** | 0.27, 1.24 | 0.71** | 0.22, 1.20 | 0.52* | 0.06, 0.98 | 0.52* | 0.06, 0.98 |
| Family affluence |  |  |  |  |  |  |  |  |
| Lowest 20ptc (ref.) | 0.00 | - | 0.00 | - | 0.00 | - | 0.00 | - |
| Medium 60ptc | 0.53 | -0.01, 1.07 | 0.51 | -0.04, 1.06 | 0.69* | 0.13, 1.25 | 0.69* | 0.13, 1.24 |
| Highest 20ptc | 0.15 | -0.51, 0.80 | 0.21 | -0.45, 0.87 | 0.54 | -0.10, 1.19 | 0.54 | -0.10, 1.19 |
| Family support |  |  |  |  |  |  |  |  |
| Low (ref.) | 0.00 |  |  |  | 0.00 | - | 0.00 | - |
| High | -2.37*** | -2.85, -1.89 |  |  | -2.35*** | -2.83, -1.87 | -2.37** | -2.87, -1.87 |
| Interaction between weight status and family support |  |  |  |  |  |  |  |  |
| Overweight*High family support |  |  |  |  |  |  | 0.10 | -1.34, 1.53 |
| Obese*High family support |  |  |  |  |  |  | 0.31 | -2.65, 3.28 |
| Wald test |  |  |  |  |  |  | p=0.972 |  |

***p<0.001 *p<0.05

Crude analyses include one variable at a time.

Model 1 includes weight status, age, and family affluence.

Model 2 includes weight status, age, family affluence, and family support.

Model 3 includes weight status, age, family affluence, family support, and the interaction between weight status and family support.

**Supplementary Material. Table S5.** Results from linear regression analyses of psychological complaints regressed on weight status and covariates, among girls. Regression coefficients and 95% confidence intervals (95% CI) with robust standard errors. n=1,607

|  | Crude | | Model 1 | | Model 2 | | Model 3 | |
| --- | --- | --- | --- | --- | --- | --- | --- | --- |
|  | b | 95% CI | b | 95% CI | b | 95% CI | b | 95% CI |
| Weight status |  |  |  |  |  |  |  |  |
| Non-overweight (ref.) | 0.00 | - | 0.00 | - | 0.00 | - | 0.00 | - |
| Overweight | 0.39 | -0.33, 1.12 | 0.40 | -0.35, 1.15 | 0.15 | -0.51, 0.81 | 0.15 | -0.95, 1.25 |
| Obese | 1.56* | 0.06, 3.07 | 1.42 | -0.03, 2.88 | 0.97 | -0.23, 2.17 | 2.49** | 1.05, 3.94 |
| Age |  |  |  |  |  |  |  |  |
| 11 years (ref.) | 0.00 | - | 0.00 | - | 0.00 | - | 0.00 | - |
| 13 years | 1.19*** | 0.59, 1.79 | 1.18*** | 0.57, 1.79 | 0.93** | 0.38, 1.49 | 0.93** | 0.37, 1.48 |
| 15 years | 2.16*** | 1.61, 2.71 | 2.16*** | 1.61, 2.71 | 1.70*** | 1.18, 2.22 | 1.69*** | 1.17, 2.22 |
| Family affluence |  |  |  |  |  |  |  |  |
| Lowest 20ptc (ref.) | 0.00 | - | 0.00 | - | 0.00 | - | 0.00 | - |
| Medium 60ptc | -0.29 | -0.91, 0.34 | -0.37 | -0.99, 0.25 | -0.17 | -0.72, 0.39 | -0.17 | -0.73, 0.39 |
| Highest 20ptc | -0.65 | -1.39, 0.08 | -0.45 | -1.19, 0.29 | 0.00 | -0.67, 0.67 | -0.02 | -0.70, 0.65 |
| Family support |  |  |  |  |  |  |  |  |
| Low (ref.) | 0.00 |  |  |  | 0.00 | - | 0.00 | - |
| High | -3.47*** | -3.91, -3.04 |  |  | -3.27*** | -3.69, -2.85 | -3.21*** | -3.66, -2.76 |
| Interaction between weight status and family support |  |  |  |  |  |  |  |  |
| Overweight*High family support |  |  |  |  |  |  | 0.00 | -1.44, 1.45 |
| Obese*High family support |  |  |  |  |  |  | -2.66* | -5.01, -0.31 |
| Wald test |  |  |  |  |  |  | p=0.083 |  |

***p<0.001 *p<0.05

Crude analyses include one variable at a time.

Model 1 includes weight status, age, and family affluence.

Model 2 includes weight status, age, family affluence, and family support.

Model 3 includes weight status, age, family affluence, family support, and the interaction between weight status and family support.

**Supplementary Material. Table S6.** Results from linear regression analyses of somatic complaints regressed on weight status and covariates, among boys. Regression coefficients and 95% confidence intervals (95% CI) with robust standard errors. n=1,528

|  | Crude | | Model 1 | | Model 2 | | Model 3 | |
| --- | --- | --- | --- | --- | --- | --- | --- | --- |
|  | b | 95% CI | b | 95% CI | b | 95% CI | b | 95% CI |
| Weight status |  |  |  |  |  |  |  |  |
| Non-overweight (ref.) | 0.00 | - | 0.00 | - | 0.00 | - | 0.00 | - |
| Overweight | 0.05 | -0.41, 0.51 | 0.05 | -0.41, 0.51 | 0.06 | -0.40, 0.51 | 0.28 | -0.86, 1.42 |
| Obese | 1.63** | 0.42, 2.84 | 1.63** | 0.43, 2.84 | 1.59** | 0.45, 2.74 | 3.88* | 0.92, 6.85 |
| Age |  |  |  |  |  |  |  |  |
| 11 years (ref.) | 0.00 | - | 0.00 | - | 0.00 | - | 0.00 | - |
| 13 years | 0.08 | -0.44, 0.60 | 0.05 | -0.47. 0.58 | 0.01 | -0.51, 0.52 | 0.01 | -0.51, 0.52 |
| 15 years | 0.41 | -0.11, 0.93 | 0.39 | -0.13, 0.92 | 0.30 | -0.22, 0.82 | 0.29 | -0.22, 0.81 |
| Family affluence |  |  |  |  |  |  |  |  |
| Lowest 20ptc (ref.) | 0.00 | - | 0.00 | - | 0.00 | - | 0.00 | - |
| Medium 60ptc | -0.03 | -0.53, 0.47 | -0.06 | -0.55, 0.43 | 0.03 | -0.46, 0.52 | 0.07 | -0.41, 0.56 |
| Highest 20ptc | -0.19 | -0.86, 0.48 | -0.16 | -0.82, 0.50 | 0.00 | -0.66, 0.66 | 0.03 | -0.63, 0.70 |
| Family support |  |  |  |  |  |  |  |  |
| Low (ref.) | 0.00 |  |  |  | 0.00 | - | 0.00 | - |
| High | -1.15*** | -1.57, -0.72 |  |  | -1.11 | -1.53, -0.69 | -1.00*** | -1.43, -0.56 |
| Interaction between weight status and family support |  |  |  |  |  |  |  |  |
| Overweight*High family support |  |  |  |  |  |  | -0.28 | -1.57, 1.00 |
| Obese*High family support |  |  |  |  |  |  | -3.05 | -6.15, 0.06 |
| Wald test |  |  |  |  |  |  | p=0.154 |  |

***p<0.001 *p<0.05

Crude analyses include one variable at a time.

Model 1 includes weight status, age, and family affluence.

Model 2 includes weight status, age, family affluence, and family support.

Model 3 includes weight status, age, family affluence, family support, and the interaction between weight status and family support.

**Supplementary Material. Table S7.** Results from linear regression analyses of somatic complaints regressed on weight status and covariates, among girls. Regression coefficients and 95% confidence intervals (95% CI) with robust standard errors. n=1,607

|  | Crude | | Model 1 | | Model 2 | | Model 3 | |
| --- | --- | --- | --- | --- | --- | --- | --- | --- |
|  | b | 95% CI | b | 95% CI | b | 95% CI | b | 95% CI |
| Weight status |  |  |  |  |  |  |  |  |
| Non-overweight (ref.) | 0.00 | - | 0.00 | - | 0.00 | - | 0.00 | - |
| Overweight | 0.39 | -0.23, 1.01 | 0.35 | -0.24, 0.95 | 0.20 | -0.38, 0.78 | 0.12 | -0.96, 1.20 |
| Obese | 1.79** | 0.53, 3.04 | 1.66* | 0.37, 2.96 | 1.38* | 0.23, 2.53 | 3.40** | 1.09, 5.72 |
| Age |  |  |  |  |  |  |  |  |
| 11 years (ref.) | 0.00 | - | 0.00 | - | 0.00 | - | 0.00 | - |
| 13 years | 0.98*** | 0.48, 1.47 | 0.97*** | 0.47, 1.47 | 0.82** | 0.36, 1.28 | 0.82** | 0.36, 1.27 |
| 15 years | 2.00*** | 1.53, 2.47 | 2.00*** | 1.53, 2.47 | 1.72*** | 1.26, 2.18 | 1.71*** | 1.26, 2.17 |
| Family affluence |  |  |  |  |  |  |  |  |
| Lowest 20ptc (ref.) | 0.00 | - | 0.00 | - | 0.00 | - | 0.00 | - |
| Medium 60ptc | -0.54* | -1.07, -0.01 | -0.61* | -1.13, -0.09 | -0.49 | -0.98, 0.00 | -0.49* | -0.98, 0.00 |
| Highest 20ptc | -0.87** | -1.50, -0.24 | -0.68* | -1.29, -0.08 | -0.41 | -.0.99, 0.17 | -0.44 | -1.02, 0.14 |
| Family support |  |  |  |  |  |  |  |  |
| Low (ref.) | 0.00 |  |  |  | 0.00 | - | 0.00 | - |
| High | -2.24*** | -2.69, -1.80 |  |  | -2.01*** | -2.45, -1.57 | -1.94*** | -2.39, -1.50 |
| Interaction between weight status and family support |  |  |  |  |  |  |  |  |
| Overweight*High family support |  |  |  |  |  |  | 0.13 | -1.23, 1.49 |
| Obese*High family support |  |  |  |  |  |  | -3.53* | -6.46, -0.60 |
| Wald test |  |  |  |  |  |  | p=0.062 |  |

***p<0.001 *p<0.05

Crude analyses include one variable at a time.

Model 1 includes weight status, age, and family affluence.

Model 2 includes weight status, age, family affluence, and family support.

Model 3 includes weight status, age, family affluence, family support, and the interaction between weight status and family support.
